# Controllable Charge Transfer in Ag-TiO_2_ Composite Structure for SERS Application

**DOI:** 10.3390/nano7070159

**Published:** 2017-06-28

**Authors:** Yaxin Wang, Chao Yan, Lei Chen, Yongjun Zhang, Jinghai Yang

**Affiliations:** 1 Key Laboratory of Functional Materials Physics and Chemistry, Jilin Normal University, Ministry of Education, Siping 136000, China; wangyaxin1010@126.com (Y.W.); 18686656043@163.com (C.Y.); chenlei@jlnu.edu.cn (L.C.); jhyang1@jlnu.edu.cn (J.Y.); 2 Key Laboratory of Excited State Physics, Changchun Institute of Optics Fine Mechanics and Physics, Chinese Academy of Sciences, Changchun 130033, China

**Keywords:** SERS, TiO_2_-Ag nanocap array, magnetron sputtering, metal-semiconductor composite

## Abstract

The nanocaps array of TiO_2_/Ag bilayer with different Ag thicknesses and co-sputtering TiO_2_-Ag monolayer with different TiO_2_ contents were fabricated on a two-dimensional colloidal array substrate for the investigation of Surface enhanced Raman scattering (SERS) properties. For the TiO_2_/Ag bilayer, when the Ag thickness increased, SERS intensity decreased. Meanwhile, a significant enhancement was observed when the sublayer Ag was 10 nm compared to the pure Ag monolayer, which was ascribed to the metal-semiconductor synergistic effect that electromagnetic mechanism (EM) provided by roughness surface and charge-transfer (CT) enhancement mechanism from TiO_2_-Ag composite components. In comparison to the TiO_2_/Ag bilayer, the co-sputtered TiO_2_-Ag monolayer decreased the aggregation of Ag particles and led to the formation of small Ag particles, which showed that TiO_2_ could effectively inhibit the aggregation and growth of Ag nanoparticles.

## 1. Introduction 

Surface enhanced Raman scattering (SERS) was initially observed by Fleischmann in 1974, and was further developed by Jeanmaire and Van Duyne in 1977 [1,2]. Nowadays, the intensity of SERS signal is greatly improved, and the enhancement factor can be as high as 10^14^ so that single molecule detection can be realized by SERS technology. SERS has been widely used in many fields, such as catalytic, biological sensing, medical detection, trace analysis, and so on [3,4,5,6]. In order to describe the enhancement mechanism in SERS, two main contributions from the electromagnetic mechanism (EM) and the charge-transfer mechanism (CT) are discussed [2,7].

The development of SERS technology and the expansion of the application field depends on the excellent performance of the substrate materials. With the discovery of metallic nanoparticles possessing SERS enhancement properties, noble metals (e.g., Ag, Au) and transition metals (e.g., Pt, Pd) have become the most studied classes of SERS-active substrates [8,9,10]. Moreover, semiconductor materials (ZnO and TiO_2_) also display plasmon resonance within visible and NIR frequencies [11,12]. In recent years, the composite substrate of noble metals and semiconductor functional materials have attracted researchers’ interests because their combination can not only improve the property of SERS substrates, but can also extend SERS substrates’ applications, in contrast to substrates consisting of a single component [13,14,15,16].

Compared with other noble metals, Ag material is widely used in SERS substrates because of its high surface plasmon resonance in the visible region that can provide great contributions to the electromagnetic field and high SERS activity. The semiconductor material TiO_2_ with a wide band gap is usually selected in many important applications areas, such as catalytic [17,18,19,20] and antimicrobial [21,22,23] applications, due to its merits including low cost, high stability, and bio-compatibility. Most SERS substrates with Ag and TiO_2_ composite components are prepared by a chemical method, such as the sol-gel method, chemical vapor deposition, and so on. However, in order to obtain a SERS substrate comprising a nanoarray with a large area and high ordered structures, a physical preparation process with simple steps is a good choice [24,25]. One such simple process is magnetron sputtering. Films prepared by magnetron sputtering have good stability and uniformity. In this paper, different nanostructure arrays consisting of TiO_2_ and Ag composites are fabricated by the magnetron sputtering system. In comparison to the pure Ag array, the properties of the nanostructure array for a TiO_2_/Ag bilayer and a TiO_2_-Ag monolayer deposited on a two-dimensional colloidal array are investigated by 4-Amino-thiophenol (PATP) as the probing molecule.

## 2. Results and Discussion

### 2.1. Preparation and Characterization of the Nano Composite Structure

We prepared the polystyrene (PS) colloidal spheres Arrays with size of 200 nm by the self-assembly technique. The Si wafer was immersed in a mixed solution containing NH_4_OH, H_2_O_2_, and H_2_O at a volume ratio of 1:2:6, and was heated at 300 °C for 5-10 min. Then, they were ultrasonically cleaned for 10–15 min in deionized water and alcohol alternately. This cleaning step was repeated three times. The Si wafer was soaked in 2% sodium sulfate solution for 24 h to obtain a hydrophilic substrate surface. Alcohol and 200 nm polystyrene were mixed with a volume ratio of 1:1. Subsequently, we dropped the mixture on the Si wafer, which was kept in a sodium dodecyl sulfate solution and slowly immersed into a glass vessel. The PS particles formed unordered monolayer films on the water surface. Finally, after absorbing excess water by filter paper and being dried completely in air by static natural evaporation, two-dimensional polystyrene beads in closely-packed monolayer ordered arrays formed on the surface of the silicon substrate.

**Figure 1:**
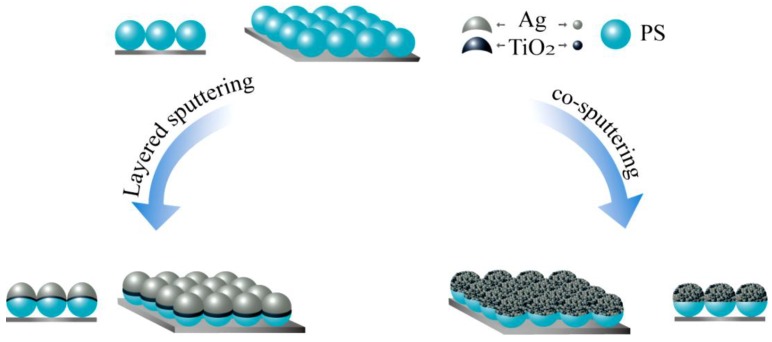
Schematic diagram of the preparation process for the nanocap arrays of the TiO_2_/Ag bilayer and the TiO_2_-Ag monolayer.

Semiconductor TiO_2_ and metal Ag targets were deposited onto 200-nm PS colloidal spheres in the magnetron sputtering system to fabricate the nanocap arrays of the TiO_2_ (10 nm)/Ag (10–40 nm) bilayer with different Ag thicknesses and the co-sputtered TiO_2_-Ag (10 nm) monolayer. The fabrication process is given in Figure 1. For comparison, TiO_2_ and Ag were alternatively sputtered and co-sputtered to form bilayer and monolayer nanocap arrays, respectively. The TiO_2_ was amorphous in the bilayer and monolayer, as shown in Figure S1. The simple and facile magnetron sputtering technology was chosen to fabricate the nanostructure array, which avoids the necessity of sophisticated steps during fabrication.

**Figure 2:**
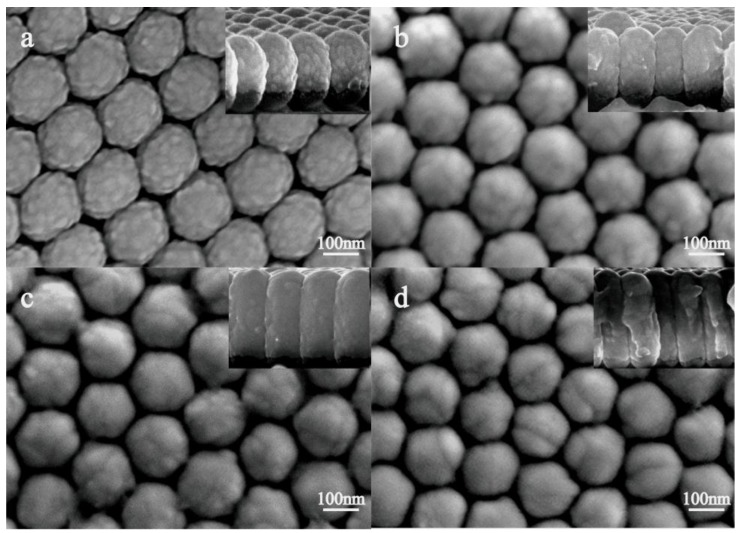
SEM images of bilayer TiO_2_ (10 nm)/Ag t nm (from (**a**) to (**d**), t = 10 nm, 20 nm, 30 nm, 40 nm) on the PS template for different thicknesses of the Ag layer. The illustration is the cross-section of the sample.

Figure 2a–d show the SEM images for the bilayer TiO_2_ (10 nm)/Ag t nm (t = 10 nm, 20 nm, 30 nm, 40 nm) on the PS template. The aggregations of particles on the surface of PS became obvious, and surface roughness decreased as the Ag thickness increased from 10 nm to 40 nm. The amplified cross-section images show that the thickness of the films increased gradually and the morphologies changed from spherical to columnar shapes. When the PS colloidal sphere was used as the template, the spherical substrates induced the film to grow in the vertical direction on the sphere surface, and the adjacent nanocaps were connected to each other when the film was very thick. Ag and TiO_2_ elements were distributed on the surface of the nanocap randomly, and the element analysis mapping of the TiO_2_ (10 nm)/Ag (10 nm)-coated nanocap surface is shown in Figure S2.

### 2.2. SERS Study of PS/TiO_2_/Ag and PS/Ag

**Figure 3:**
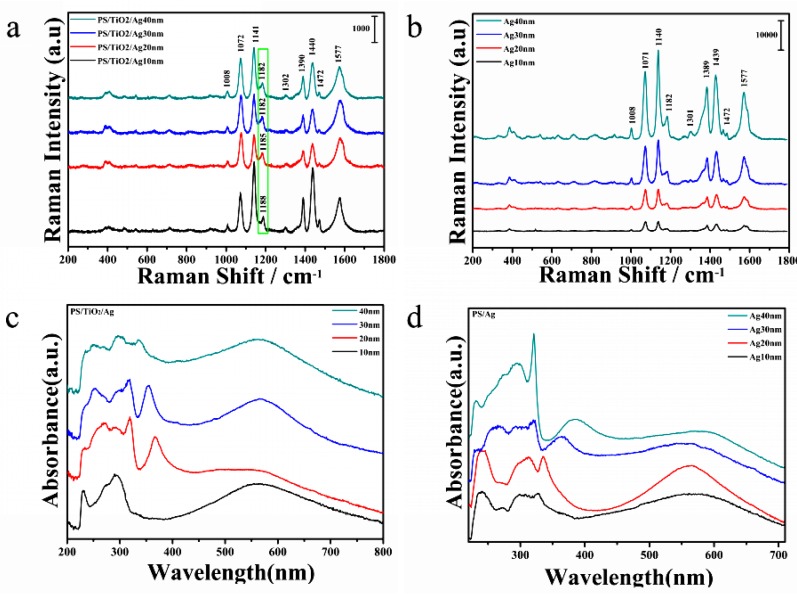
SERS spectra of (**a**) PS/TiO_2_ (10 nm) /Ag (10–40 nm) bilayer; (**b**) PS/Ag (10–40 nm) monolayer resembled on 200 nm PS template; (**c**) UV-Vis absorption spectra of PS/TiO_2_ (10 nm)/Ag (10–40 nm) bilayer; (**d**) UV-Vis absorption spectra of PS/Ag (10–40 nm).

Figure 3 shows the SERS spectrum of PATP adsorbed on the PS/TiO_2_/Ag bilayer from 1 × 10^−3^ mol/L aqueous solution. For the substrate PS/TiO_2_/Ag bilayer and PS/Ag monolayer, the relative intensity and the position of Raman peaks are significantly different. In the PS/TiO_2_/Ag spectra (Figure 3a), PATP molecules exhibit characteristic peaks located at 1008, 1072, 1141, 1188, 1302, 1390, 1440, 1472, and 1577 cm^−1^. However, PATP molecules on the PS/Ag substrate show the characteristic peaks located at 1008, 1071, 1140, 1182, 1301, 1389, 1439, 1472, and 1577 cm^−1^ (Figure 3b). Among these, the peaks at around 1072, 1188, and 1472 cm^−1^ are assigned to the υ(C–S), δ(C–H), and υ(C–C) stretching vibrations, respectively, which are dominated by characteristic a_1_ vibrational modes [26,27,28]. The δ(C–H) at 1140 cm^−1^, [υ(C–C) + δ(C–H)] at 1390 and 1440 cm^−1^, and υ(C–C) at 1577 cm^−1^ are interpreted as b_2_ modes [29,30,31] (Table 1).

**Figure 4:**
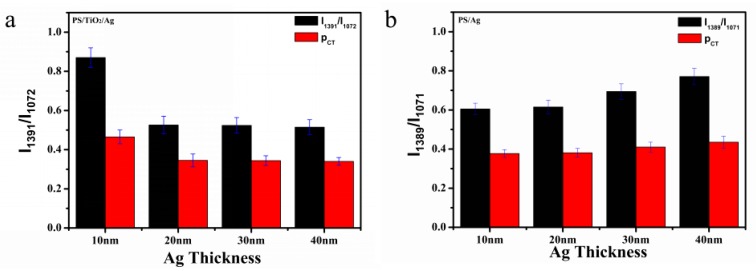
Column statistics of relative peak intensity and charge transfer for (**a**) I_1391_/I_1072_ of the PS/TiO_2_ (10 nm)/Ag (10–40 nm) bilayer and (**b**) I_1389_/I_1071_ of the pure PS/Ag (10–40 nm) monolayer.

In the UV-Vis spectra of the TiO_2_/Ag bilayer (Figure 3c), two major absorption bands are observed. The band around 200–400 nm corresponds to the band gap absorption of TiO_2_-Ag, while the varying around 400–800 nm is attributed to the surface plasmon resonance (SPR) absorption of Ag. The blue shift of absorption peaks and the decrease of resonance intensity can be observed when the Ag thickness increases from 10 nm to 40 nm, which can be ascribed to the reconstruction of a new Fermi level in TiO_2_-Ag bilayer due to the special optical properties of TiO_2_ that can provide a large number of electrons [32]. The peak changes in the TiO_2_/Ag bilayer should be derived from the interaction between the chemical absorption of the PATP molecule and the nanostructure of the TiO_2_/Ag bilayer. Therefore, the charge transfer (CT) is calculated for the TiO_2_/Ag bilayer and pure Ag monolayer arrays, and the results are shown in Figure 4. Figure 3d presents the UV-Vis spectra of the as-prepared pure Ag nanomaterials with four different thicknesses of Ag. The broad absorption covering the range of 400–800 nm should be ascribed to the SPR of Ag. The red shift phenomenon appears when the thickness of Ag increases. The wavelength of the SPR band is related to the particle size, shape, and surrounding environment [33,34]. In addition, the spectra of Ag 40 nm show a sharp rise at about 320 nm, which corresponds to the onset of the interband absorption threshold energy hv = 3.9 eV of Ag [32]. In contrast to the Ag monolayer, the characteristic peaks exhibited are partly in the blue shift in the TiO_2_/Ag bilayer. For the TiO_2_/Ag bilayer, the SERS intensities decreased with the increase of Ag thickness, and the maximum value is observed when the thickness of the sublayer Ag is 10 nm, while the sample shows the weakest signal when the sublayer of Ag is 40 nm. However, for the Ag monolayer array, the intensity of the SERS signals increases with theincrease of the Ag thickness, and the maximum value is observed for an Ag thickness of 40 nm. Furthermore, with the increase of the content of Ag, the 1188 cm^−1^ characteristic peaks of PATP molecules on TiO_2_/Ag bilayer exhibited the blue shift from 1188 cm^−1^ of TiO_2_/Ag (10 nm) to 1182 cm^−1^ for the sample TiO_2_/Ag (40 nm) (Figure 4a). For the pure Ag monolayer (Figure 3b), the peaks do not show any shift. The above results indicate that TiO_2_ plays an important role in the peak shifts of PATP molecules absorbed on the TiO_2_/Ag bilayer. As TiO_2_ and Ag form composites, the size of nanoparticles changes. The peak shifts for TiO_2_/Ag can be reduced when the thickness of the Ag layer increases.

The band at the 1072 cm^−1^ (a_1_) peak of the C–S stretching vibration and the band at about 1391 cm^−1^ (b_2_) of the C–H and C–C bending vibrations are used to calculate the charge transfer degree. It is clear that the ratio of relative intensities of the two Raman peaks (1391 cm^−1^ and 1072 cm^−1^) of PATP molecules adsorbed on TiO_2_/Ag bilayer is remarkably changed. The charge transfer of the bilayer decreases upon increasing the thickness of the Ag layer, which indicates that the TiO_2_ on the surface of the bilayer plays an important role in charge transfer. We believe that the mixture components consisting of Ag and TiO_2_ occur at the surface of the bilayer due to the interface diffusion between the TiO_2_ and Ag sublayer when a thinner Ag film is deposited on TiO_2_. Furthermore, TiO_2_ can provide a large number of electrons due to its unique photoelectric properties and generate a new Fermi level in the composite structure, which causes a change in the charge transfer degree between the composites and PATP molecules. In general, the changes in the relative Raman band intensities between different vibrational modes are closely related to the chemical mechanism of SERS [35].

We use the concept of the “degree of CT (ρCT)”, proposed by Lombardi et al. [36,37] to estimate the influence of the contribution of CT resonance to the SERS intensity quantitatively. The ρCT(k) can be determined by following equation:

(1) $$\rho \mathrm{CT}(k)=\frac{I^{k}(CT)-I^{k}(SPR)}{I^{k}(CT)+I^{0}(SPR)}$$

Here, *k* is an index used to identify individual molecular lines in the Raman spectrum. We choose the intensities of two peaks (1072 cm^−1^ and 1391 cm^−1^) in a spectral region for better understanding. The 1072 cm^−1^ (a_1_) is totally symmetric to the SERS signal contributions from SPR, whose intensity is denoted as $I^{0}(SPR)$, and for this line $I^{k}(SPR)=I^{0}(SPR)$. The other peak, 1391 cm^−1^ (b_2_), is non-totally symmetric (intensity denoted as $I^{k}(CT)$). It is the measured intensity in the region of the spectrum where CT resonance makes an additional contribution to the SERS intensity, excluding the contribution of SPR [31]. In this case, $I^{k}(SPR)$ is normally small or equal to zero. Then, Equation (2) can be approximately expressed as follows:

(2) $$\rho CT=\frac{\frac{b_{2}}{a_{1}}}{1+\frac{b_{2}}{a_{1}}}$$

In Figure 4a, the change of relative peak intensities of I_1391_/I_1072_ vibrational mode bands indicates the CT transition from the Fermi level of the TiO_2_-Ag composites to the lowest unoccupied molecular orbitals (LUMO) of the PATP molecules. With an increasing concentration of Ag, the charges of the TiO_2_-Ag-PATP complex can be redistributed further, resulting in a more suitable CT state involved in the SERS-CT process. The degree of CT (ρCT) shows the match degree of excitation energy and the energy difference between the Fermi level of the TiO_2_-Ag composites and the LUMO of PATP, indicating that the b_2_ mode of PATP is selectively enhanced via the CT resonance transition. However, in the pure Ag monolayer (Figure 4b), Ag provides a large number of electrons in the charge transfer process so that more free electrons can be provided, which makes the charge transfer degree increase with the increase in Ag content.

### 2.3. Enhancement Factor (EF) of PS/TiO_2_/Ag and PS/TiO_2_:Ag

**Figure 5:**
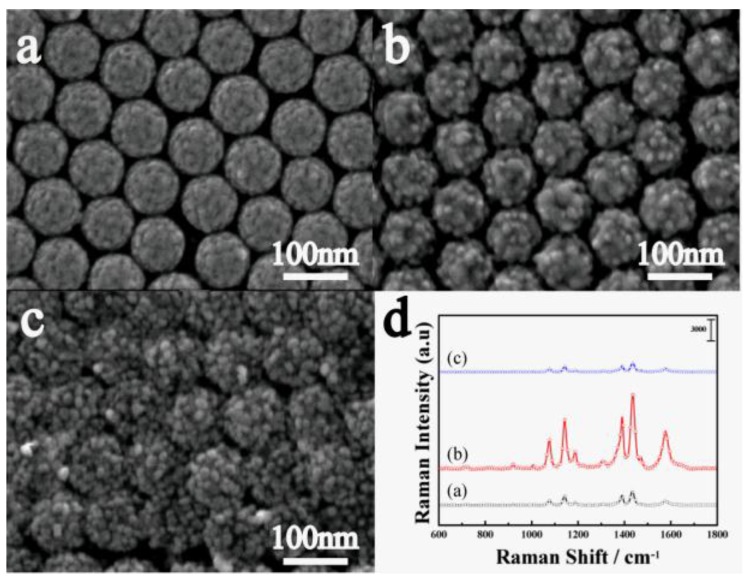
(**a**–**c**) SEM images and (**d**) SERS spectra of TiO_2_-Ag (20 nm) with different TiO_2_ atomic percent contents: (**a**) 10%; (**b**) 15%; (**c**) 25%.

**Figure 6:**
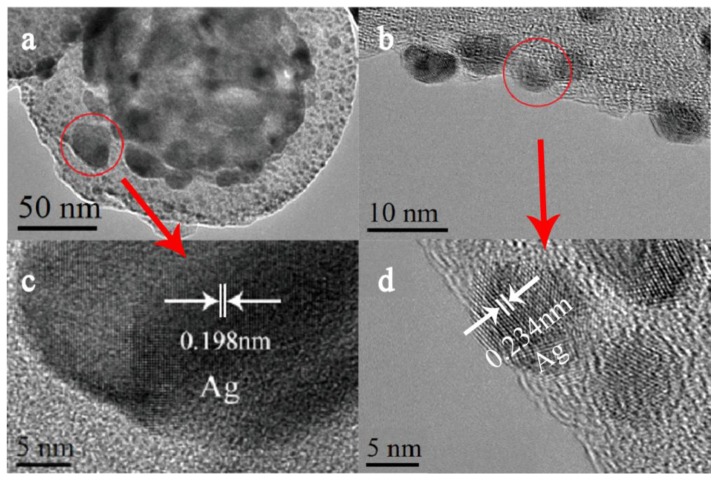
TEM and HRTEM images of (**a**,**c**) the TiO_2_ (10 nm)/Ag (10 nm) bilayer, and (**b**,**d**) the co-sputtered TiO_2_-Ag (20 nm) monolayer.

Except for the charge transfer, the change of the surface morphology of nanocaps array is another important factor for the enhancement of SERS for PS/TiO_2_ (10 nm)/Ag (10 nm) bilayer. As the SEM image of Figure 2 shows, a 10-nm Ag film was deposited on TiO_2_, and the composite of Ag and TiO_2_ increased the surface roughness, leading to the enhancement of the SERS signal. When the TiO_2_ layer was covered completely by the Ag film, the SERS signals decreased with the increase of the Ag thickness. To investigate the effect of the surface roughness and composite consisting of TiO_2_ and Ag on SERS, the co-sputtered TiO_2_-Ag (20 nm) monolayer was deposited on a PS template. Figure 5a–d show the SEM images and SERS spectra of the TiO_2_-Ag monolayer with different contents of TiO_2_. When the Ti content increased from 10 at.% to 28 at.%, the particles sizes and the surface roughness increased. The SERS intensity increased first and then decreased. Also, the maximum occurred when the TiO_2_ atomic percent content was 15%, as it was confirmed that the optimum surface roughness provided abundant hot spots that were created by the composite of TiO_2_ and Ag. Compared to the bilayer samples, TEM images show the small Ag particles formed in the co-sputtered monolayer (Figure 6). In addition, HRTEM images show that TiO_2_ is amorphous and the Ag particles trapped in amorphous TiO_2_ are about 8 nm, which is smaller than that of the bilayer of 30 nm, which shows that co-sputtered TiO_2_ can effectively inhibit the aggregation and growth of Ag nanoparticles.

**Figure 7:**
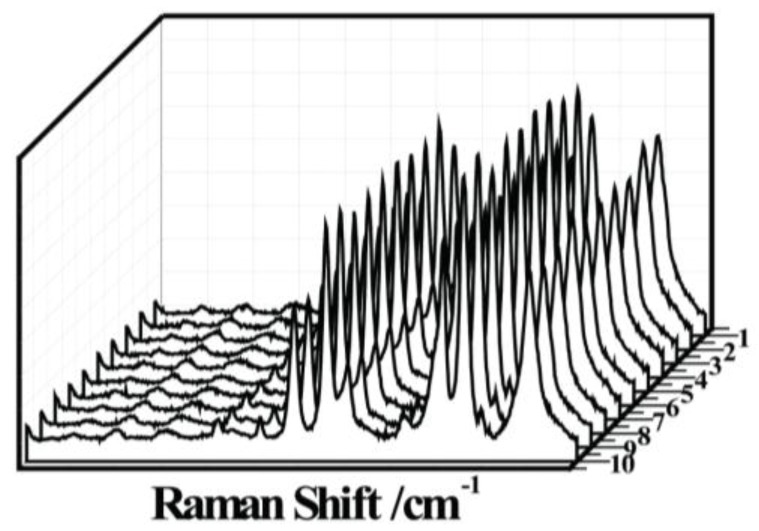
Reproducibility test for SERS spectra of the TiO_2_ (10 nm)/Ag (10 nm) bilayer.

The SERS enhancement factor (EF) is calculated from 10 randomly selected points for the TiO_2_ (10 nm)/Ag (10 nm) bilayer and TiO_2_-Ag (20 nm) co-sputtered monolayer according to the equation [38,39,40] (Figure 7), $EF=(I_{SERS}\times N_{bulk})/(I_{bulk}\times N_{ads})$, where $I_{SERS}$ and $I_{bulk}$ are the SERS intensity of the bands at 1440 cm^−1^ assigned to PATP absorbed on TiO_2_/Ag arrays and the Raman intensity of the band at 1403 cm^−1^ assigned to the solid PATP, respectively. $N_{bulk}=cRSVN_{A}$ is the average number of molecules in the scattering volume (V) for the Raman (non-SERS) measurement. The molar concentration (cRS) of the PATP analyte on the reference region is 1 mM. $N_{ads}$ is the average number of adsorbed molecules in the scattering volume for the SERS experiments. $N_{ads}=N_{d}A_{laser}A_{N}/\delta$, where $N_{d}$ is the number density of PS with a diameter of 200 nm, and $A_{laser}$ is the area of the focal laser spot. The laser spot is a circle with a diameter of 1 μm. $A_{N}$ is the half surface area of one PS with a diameter of 200 nm, and *δ* is the surface area occupied by a single PATP adsorbed on the substrate value, which is estimated to be 0.20 nm^2^ [41]. For the Renishaw Micro-Raman spectrometer with 633-nm laser excitation, the effective focused depth is 19 μm. $N_{bulk}$ and $N_{ads}$ can be calculated to be 1.77 × 10^16^ and 9.62 × 10^7^, respectively. $I_{SERS}/I_{bulk}$ are 261.3 and 17.23 for the TiO_2_/Ag bilayer and the TiO_2_-Ag co-sputtered monolayer at the band of 1391 cm^−1^, respectively. Therefore, EF is calculated to be 4.81 × 10^10^ and 3.17 × 10^9^ for the TiO_2_/Ag bilayer and the TiO_2_-Ag co-sputtered monolayer, respectively. These results show that the monolayer prepared by the co-sputtering method has better homogeneity and SERS property than those of the bilayer.

## 3. Materials and Methods

4-Aminothiophenol (PATP) with a purity of 99.9% was used as the probing molecule. Sodium dodecyl sulfate and ethanol were purchased from Sigma Aldrich (St. Louis, MO, USA). Concentration of 10 wt % of (particle deviation less than 10%) monodisperse polystyrene (PS) colloidal spheres with a size of 200 nm were purchased from The Duke (Durham, NC, USA). The Ag and TiO_2_ targets with a purity of 99.99% (wt %) used in the experiment were purchased from Beijing TIANRY Science & Technology Developing Center (Beijing, China). Silicon wafer was purchased from Hefei Kejing Materials Technology Co., Ltd (Hefei, China). Ultrapure water (18.0 MΩ·cm^−1^) was used throughout the present study.

The film deposition was performed in a magnetron sputtering system with JGP-560C. During deposition, the base vacuum degree was 2 × 10^−4^ Pa, and the argon pressure was 0.6 Pa. The sputtering powers of TiO_2_ were 50 W, 100 W, and 150 W; the sputtering power of Ag was 5.4 W. The PS (200 nm) monolayer colloidal array was prepared by using the self-assembly method [42]. The samples were immersed in PATP solution with a concentration of 3% for more than 30 min to reach saturation adsorption. The morphology and microstructure of the samples were investigated by field emission scanning electron microscopy (SEM) and electron microscopy (TEM). The SEM images were characterized under an accelerating voltage of 5.0 KV (model JSM-7800F, JEOL, Tokyo, Japan). TEM images were recorded on JEM-2100HR (JEOL, Tokyo, Japan). UV-Vis spectra were measured on a Shimadzu UV-3600 spectrophotometer (Shimadzu, Kyoto, Japan). Raman spectra were obtained with a Renishaw Raman system model 2000 confocal microscopy spectrometer (Renishaw, London, UK). An air-cooled argon ion laser (Renishaw, London, UK) with 633-nm radiation from (40 mW, power out of 0.1%) was used for the SERS. The spectra were recorded with an accumulation time of 10 s. Film thickness was measured by a Stylus Profiler (Vecco Dektak 150, VEECO, Plainview, NY, USA).

## 4. Conclusions

In summary, we fabricated SERS substrate of TiO_2_ and Ag metal-semiconductor composite onto a 200-nm PS template by using a combination of the self-assembly technique and the magnetron sputtering method. Compared with the pure Ag film, the TiO_2_/Ag bilayer showed significant SERS enhancement when the Ag thickness was thin, which was ascribed to the electromagnetic mechanism (EM) and charge-transfer (CT) enhancement mechanisms in the metal-semiconductor system. The optimized TiO_2_ contents at the surface of the nanostructure not only improved the enhancement of SERS but also effectively inhibited the aggregation and growth of Ag nanoparticles, which was confirmed by the co-sputtered TiO_2_-Ag monolayer. The TiO_2_-Ag nanocaps array exhibited a good periodicity and uniformity; the enhancement factor reached 4.81 × 10^10^ for the TiO_2_/Ag bilayer and 3.17 × 10^9^ for the TiO_2_-Ag monolayer, respectively.

## Figures and Tables

**Table 1:** Wavenumbers and assignments of bands in the SERS spectrum of PATP molecule.

Wavenumber (cm^−1^)		Band Assignment
PS/TiO_2_/Ag	PS/Ag	
1577m	1577m	υCC, 8b(b_2_)
1472w	1472w	υCC, 19a(a_1_)
1440υs	1439υs	υCC + δCH, 19b(b_2_)
1390s	1389s	δCH + υCC, 3(b_2_)
1302w	1301w	υCC + δCH, 14(b_2_)
1188w	1182w	δCH, 9a(a_1_)
1141υs	1140υs	δCH, 9b(b_2_)
1072m	1071m	υCS, 7a(a_1_)
1008w	1008w	γCC + γCCC, 18a(a_1_)

Approximate description of the modes (υ, stretch; δ and γ, bend). Frequencies (in cm^−1^) followed by relative intensities (υs, very strong; s, strong; m, medium; w, weak).

## Supplementary Materials

The following are available online at http://www.mdpi.com/2079-4991/7/7/159/s1, Figure S1: XRD patterns of samples for TiO_2_/Ag bilayer and monolayer TiO_2_ deposited on PS200 nm templates; Figure S2: The element analysis mapping of bilayer TiO_2_ (10 nm)/Ag (10 nm) nanocap arrays by SEM; Figure S3: SEM of PS200 nm template.
