# Supplementary material for: Controllable Charge Transfer in Ag-TiO2 Composite Structure for SERS Application
Source: Nanomaterials (Basel). 2017 Jun 28;7(7):159. doi: 10.3390/nano7070159 (PMC5535225; doi:10.3390/nano7070159)
Supplement: Supplementary file 1 [file nanomaterials-07-00159-s001.pdf]

Controllable charge transfer in Ag-TiO_2 composite structure for SERS application

Yaxin Wang[1], Chao Yan[1], Fan Zhang[1], Shuang Guo[1], Renxian Gao[1], Lei Chen[1], Yang Liu[1], Yongjun Zhang[1,*], Jinghai Yang[1,2]

[1] Key Laboratory of Functional Materials Physics and Chemistry, Jilin Normal University, Ministry of Education, Siping136000, PR China;
[2] Key Laboratory of Excited State Physics, Changchun Institute of Optics Fine Mechanics and Physics, Chinese Academy of Sciences, Changchun130033,PR China;
* Correspondence: E-mail: yjzhang@jlnu.edu.cn;

Academic Editor: Dr. Yuying Zhang and Dr. Wei Xie
Received: date; Accepted: date; Published: date

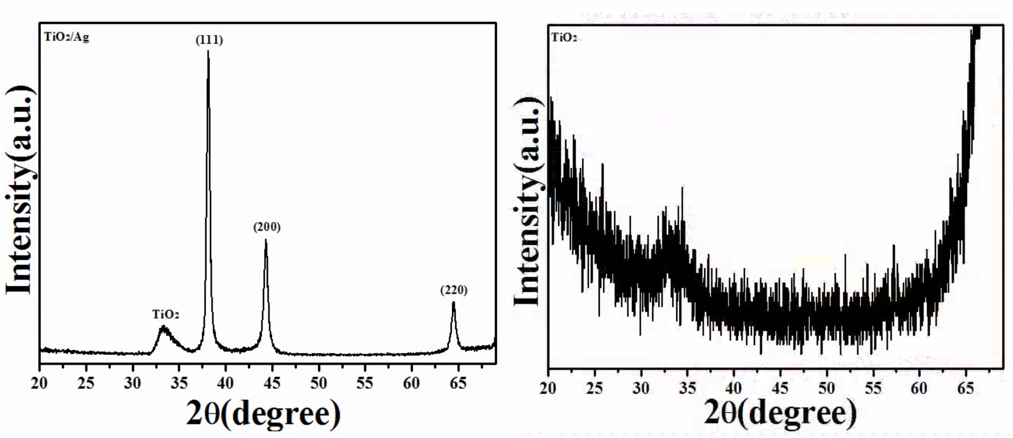

Figure S1: XRD pattern of TiO_2/Ag bilayr and monolayer TiO_2 deposited on PS200 nm template

In figure S1, the diffraction peaks at 38.1°, 44.2° and 64.4° correspond to the (1 1 1), (2 0 0) and (2 2 0) lattices in Ag with face-centered cubic structure according to JCPDS card No.04-0783, respectively. However, the broadened peak about 33° indicates titanium dioxide is amorphous, which is also confirmed by the XRD pattern of amorphous TiO_2.

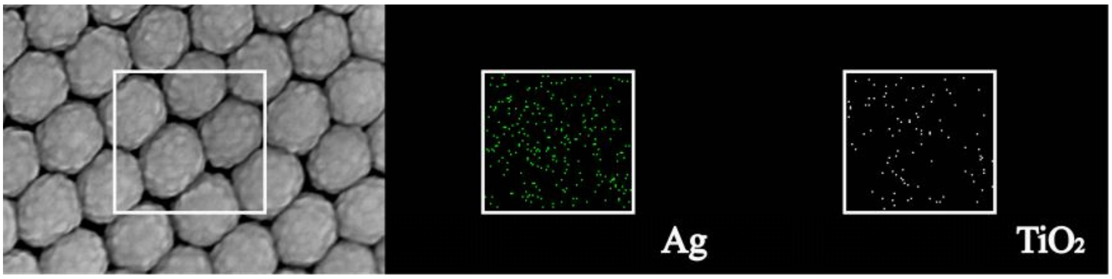

Figure S2: The element analysis mapping of bilayer TiO_2 (10 nm) /Ag (10 nm) nanocap arrays by SEM. The green and pink colors are corresponding to Ag and TiO_2 composition respectively

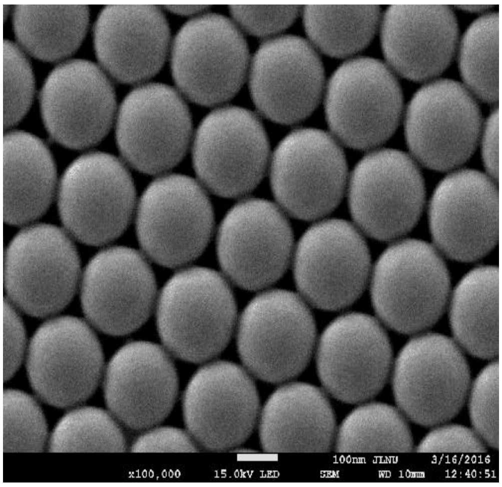

Figure S3: SEM of PS200 nm template
